# Perioperative outcomes of coronary artery bypass graft surgery in Johannesburg, South Africa

**DOI:** 10.1186/s13019-020-01385-8

**Published:** 2021-01-07

**Authors:** Samantha Reiche, Dineo Mpanya, Katharina Vanderdonck, Shungu Mogaladi, Palesa Motshabi-Chakane, Nqoba Tsabedze

**Affiliations:** 1 Division of Cardiology, Department of Internal Medicine, Faculty of Health Sciences, School of Clinical Medicine, University of the Witwatersrand, Charlotte Maxeke Johannesburg Academic Hospital, 17 Jubilee Road, Parktown, Johannesburg, 2193 South Africa; 2 Division of Cardiothoracic Surgery, Department of Surgery, Faculty of Health Sciences, School of Clinical Medicine, University of the Witwatersrand and the Charlotte Maxeke Johannesburg Academic Hospital, Johannesburg, South Africa; 3 Department of Anaesthesiology, Faculty of Health Sciences, School of Clinical Medicine, University of the Witwatersrand and the Charlotte Maxeke Johannesburg Academic Hospital, Johannesburg, South Africa

**Keywords:** Coronary artery disease, Coronary artery bypass graft surgery, Sub-Saharan Africa, Perioperative complications, Mortality

## Abstract

**Background:**

The perioperative complications in patients with coronary artery disease undergoing coronary artery bypass graft (CABG) surgery have been reported predominantly from developed countries, with a paucity of data from sub-Saharan Africa. We aim to report on the clinical characteristics and perioperative complications in patients with obstructive coronary artery disease, managed with CABG surgery at a tertiary academic hospital in Johannesburg, South Africa.

**Methods:**

We retrospectively reviewed data from adult patients who underwent CABG surgery during a 17-year period (January 2000 – December 2017). Data was collected from the cardiothoracic surgery department’s pre- and postoperative reports, the cardiology department’s medical records, and anaesthesiology’s intra-operative reports. We collected demographic, biochemical, clinical, surgical, echocardiographic, and angiographic data. Outcomes data collected included perioperative complications and mortality.

**Results:**

We analysed 1218 consecutive patient records. The study cohort consisted of 951 (78.1%) males, and the mean age was 60.1 ± 10.1 years. During the study period, 137 (11.2%) patients demised with cardiac and sepsis-related causes of death accounting for 49.6 and 37.2%, respectively. Other perioperative complications included excessive bleeding in 222 (18.2%), prolonged ventilation (exceeding 48 h) in 139 (11.4%), and sternal sepsis in 125 (10.3%). On univariate logistic regression analysis, advanced age, a lower left ventricular ejection fraction, smoking, increased cardiopulmonary bypass (CPB) time, and a higher European System for Cardiac Operative Risk Evaluation (EuroSCORE) II were all significantly associated with mortality. The EuroSCORE II [OR: 0.15 95%CI: 0.09–0.22; *p* = 0.000], and prolonged CPB time [OR: 0.01 CI: 0.00–0.02; *p* = 0.000] were independent predictors of in-hospital all-cause mortality.

**Conclusions:**

In our study, the crude perioperative mortality rate was 11.2%. Our mortality rate was significantly higher than the mortality rates reported in other developed and developing countries. To better understand the factors driving this high mortality rate, a prospective outcomes registry has been initiated, and this promises to inform on our contemporary mortality and morbidity outcomes.

## Background

The prevalence of coronary artery disease (CAD) is rapidly growing in low and middle-income countries (LMIC) [1]. Urbanisation and the adoption of a westernised diet and lifestyle are factors associated with an increased cardiovascular disease risk [2, 3]. Coronary artery bypass graft (CABG) surgery is one of the widely accepted traditional revascularisation strategies for CAD [4]. Despite the increasing number of CABG surgical procedures, there is a lack of data from sub-Saharan Africa reporting on the perioperative outcomes of patients with ischaemic heart disease undergoing CABG surgery [5]. We aim to report on the clinical characteristics and perioperative complications in patients with obstructive coronary artery disease, managed with CABG surgery in a state, academic hospital in Johannesburg, South Africa.

## Methods

We conducted a retrospective review of medical, surgical, and intensive care records of consecutive adult patients aged 18 years and older, with obstructive coronary artery disease who underwent CABG surgery at the Charlotte Maxeke Johannesburg Academic Hospital (CMJAH) between 1 January 2000 and 31 December 2017. Data was collected from the cardiothoracic surgery department’s pre- and postoperative reports, the cardiology department’s medical records, and anaesthesiology’s intra-operative reports. We collected demographic, clinical, biochemical, surgical, echocardiographic, and angiographic data.

Induction of anaesthesia was performed with full monitoring using electrocardiography, pulse oximetry, invasive arterial blood pressure, and central venous pressure monitoring. Opioid-based agents, either fentanyl 0.1 mg/kg or 2.5 μg/kg sufentanil, with midazolam 0.05 mg/kg or 0.2 mg/kg etomidate were administered. Rocuronium 1 mg/kg was typically administered for muscle relaxation. After endotracheal intubation, maintenance of anaesthesia was achieved with a volatile anaesthetic (1–2% sevoflurane end-tidal concentration). During cardiopulmonary bypass, patients received continuous infusions of an opioid (remifentanil/fentanyl/sufentanyl) and propofol. We routinely cooled patients to temperatures between 28 and 32 °C depending on the complexity of the procedure.

Blood cardioplegia was mixed as four parts of blood for one part of a crystalloid solution (Medsol). The crystalloid solution was initially administered as an induction solution higher in potassium and a maintenance solution with a lower potassium content. In each 500 ml bag of crystalloid solution (induction and maintenance), 35 ml of 50% glucose and 2 g of magnesium were added.

Immediately after cross-clamping the aorta, an induction dose of blood cardioplegia was given at 20 ml/kg. An entire induction dose was administered antegrade in some instances, but most surgeons gave 2/3rd of the induction dose antegrade into the aortic root and a third of the dose retrograde into the coronary sinus. The maintenance solution was used at 10 ml/kg or half of the induction dose for the subsequent doses. The maintenance doses were routinely administered retrograde. Also, as part of the maintenance doses, blood cardioplegia was flushed into each newly anastomosed graft. If the cross-clamp time was long, a dose of antegrade blood cardioplegia was repeatedly administered with the maintenance solution. The ischaemic time between doses of cardioplegia did not exceed 20 min.

Postoperatively, all patients were admitted to the cardiothoracic intensive care unit (ICU) until stable enough to be referred to the general cardiothoracic ward. The perioperative period was defined as the total period from admission to discharge post CABG surgery. Data on perioperative outcomes and complications was collected from surgical operative notes, ICU charts, and in-patient files. Variables collected included postoperative haemodynamic status, ventilation requirements, and complications. The European System for Cardiac Operative Risk Evaluation (EuroSCORE) II was calculated using an online calculator [6]. The EuroSCORE II features 18 variables describing patient, cardiac, and cardiac operation-related factors for predicting the risk of 30-day mortality after cardiac surgery [7]. The Research Electronic Data Capture (REDCap) tool hosted at the University of the Witwatersrand was used as the data collection and management tool.

### Statistical analysis

Continuous variables are summarised as the mean and standard deviation (SD) when normally distributed and as a median and interquartile range (IQR) when the distribution is skewed. We compared normally distributed continuous variables by using the Student t-test. The Wilcoxon rank-sum (Mann-Whitney) test was used to compare medians for non-normal data. Categorical variables are expressed as numbers and percentages. All categorical variables were compared for the study outcome by using the Pearson’s chi-square test. Odds ratios (OR) are presented with their 95% confidence interval (CI). A *p*-value of less than 0.05 was considered statistically significant. Univariable and multivariable logistic regression analyses were performed to assess for predictors of all-cause in-hospital mortality. We used STATA MP version 13.1 (StataCorp, Texas) for the statistical analysis.

## Results

**Fig. 1:**
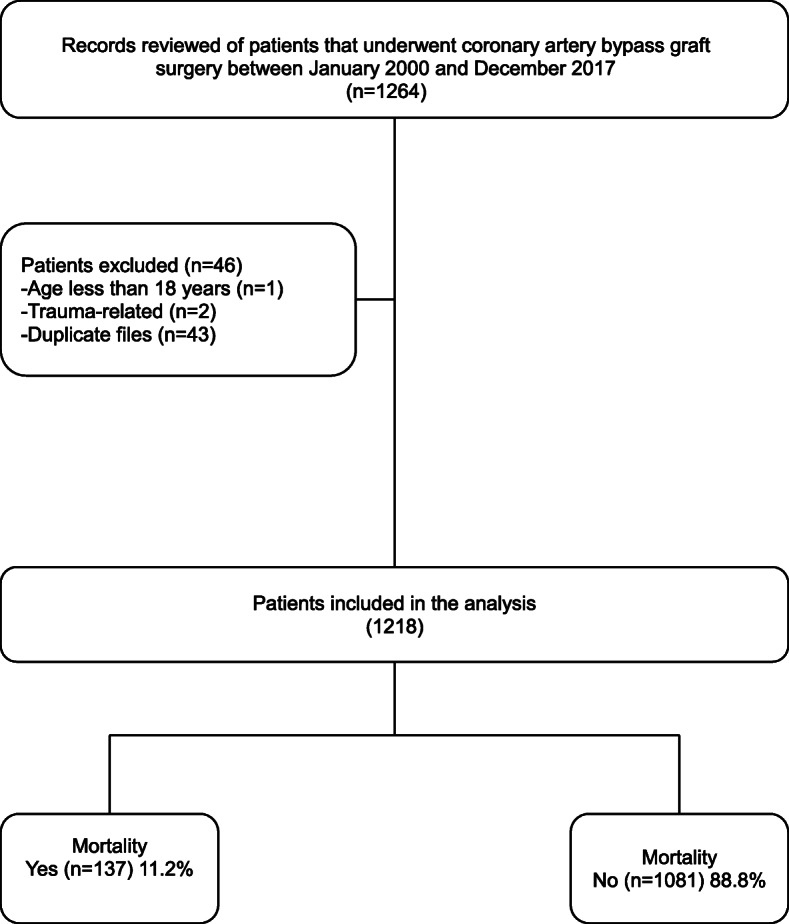
Study flow chart

**Fig. 2:**
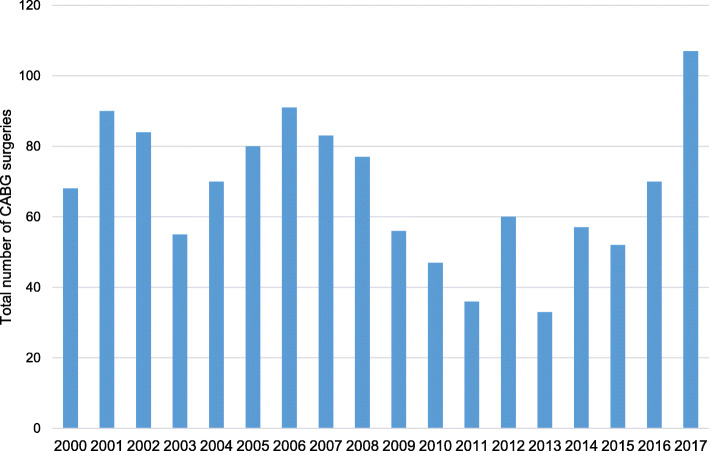
Number of coronary artery bypass graft surgeries performed between years 2000 and 2017

A total of 1218 patients were included in the analysis (Fig. 1). During the study period, the total number of CABG surgeries performed per annum varied from year to year, with less than 100 operations performed yearly on average (Fig. 2). There were 951 (78.1%) males, and the mean age in the study population was 60.1 ± 10.1 years. Hypertension, dyslipidaemia, and diabetes were the most common comorbidities reported in 74.5%, 71.0%, and 32.8% of patients, respectively. The rest of the perioperative demographic and clinical variables are summarised in Table 1.

**Table 1:** Baseline characteristics of patients referred for coronary artery bypass grafting

	All Patients (*n* = 1218)	Mortality		
		No (*n* = 1081)	Yes (*n* = 137)	*p-* value
Age, years (SD)	60.1 ± 10.1	59.6 ± 10.1	63.7 ± 9.7	0.000
Male, n (%)	951 (78.1)	844 (78.1)	107 (78.1)	0.994
Ethnicity, n (%)				0.344
White	634 (52.0)	569 (52.6)	65 (47.4)	
Indian	358 (29.4)	319 (29.5)	39 (28.5)	
Black	137 (11.2)	117 (10.8)	20 (14.6)	
Mixed ancestry	79 (6.5)	66 (6.1)	13 (9.5)	
Medical history, n (%)				
Hypertension	908 (74.5)	813 (75.2)	95 (69.3)	0.138
Diabetes mellitus	400 (32.8)	345 (31.9)	55 (40.1)	0.053
Dyslipidaemia	865 (71.0)	791 (73.2)	74 (54.0)	0.000
Stroke	32 (2.6)	27 (2.5)	5 (3.6)	0.427
PVD	50 (4.1)	42 (3.9)	8 (5.8)	0.277
COPD	159 (13.0)	138 (12.8)	21 (15.3)	0.402
Smoking	629 (51.6)	576 (53.3)	53 (38.7)	0.001
NYHA class, n (%)				0.000
I	523 (48.1)	491 (50.7)	32 (26.9)	
II	396 (36.4)	344 (35.5)	52 (43.7)	
III	141 (13.0)	118 (12.2)	23 (19.3)	
IV	27 (2.5)	15 (1.5)	12 (10.1)	
Laboratory indices				
Haemoglobin, g/dL	14.4 ± 1.7	14.5 ± 1.7	14.2 ± 1.5	0.107
Sodium, mmol/l	140 ± 3.8	140 ± 3.7	140 ± 4.8	0.107
eGFR, ml/min	86 (71–99)	87 (73–101)	74 (56–89)	0.000
LVEF, %	50.3 ± 12.8	51.2 ± 12.2	42.9 ± 14.8	0.000
CPB time (min)	139 (115–167)	137 (114–163)	162 (126–211)	0.000
Aortic clamp time (min)	89 (71–107)	89 (70–106)	95 (72–112)	0.107
EuroSCORE II	1.6 (0.9–3.5)	1.5 (0.9–3.1)	5.7 (2.5–10.9)	0.000

Data shown as mean ± standard deviation (SD) and median and interquartile ranges for continuous variables with a normal and skewed distribution, respectively. Categorical variables are summarized as absolute numbers and percentages. *COPD* Chronic obstructive pulmonary disease; *CPB* Cardiopulmonary bypass; *eGFR* Estimated glomerular filtration rate; *EuroSCORE* European system for cardiac operative risk evaluation; *LVEF* Left ventricular ejection fraction; *NYHA* New York Heart Association; *PVD* Peripheral vascular disease

The preoperative coronary angiogram demonstrated single, double, and triple vessel disease in 72 (5.9%), 238 (19.5%), and 885 (72.7%) patients, respectively. Left main stem disease was noted in 268 (22.0%) patients, and coronary artery bypass graft surgery was performed as a semi-urgent or elective procedure in 81.0% of patients. The saphenous vein was grafted in 1074 (88.2%), the left and right internal mammary artery in 1059 (86.9%) and 82 (6.7%) patients, respectively. Only 123 (10.1%) patients had grafts other than internal mammary artery grafts. The number of grafts did not differ significantly between the survivors and non-survivors [3 (IQR: 2–4), *p* = 0.405].

Throughout the study period, crystalloid cardioplegia was administered in only three patients. During the induction of cardiac arrest, the route of blood cardioplegia administration was antegrade in 89.2%, and for maintenance, antegrade infusions were used in 27.6%. In order to augment myocardial perfusion in diastole, the intra-aortic balloon pump was utilised as a cardiac assist device in 424 (37.5%) patients and was inserted before surgery in 386 (31.7%) and postoperatively in 51 (4.2%) patients. The most common indications for insertion of an intra-aortic balloon pump were left ventricular dysfunction (13.8%), followed by left main stem disease (10.3%), myocardial infarction (7.8%), cardiogenic shock (1.1%), and for catheterisation laboratory related complications in 0.7%.

During the perioperative period, 137 (11.2%) patients demised. Of these patients, cardiac-related deaths were reported in 68 (49.6%) patients, 51 (37.2%) had sepsis, 11 (8.0%) had pulmonary-related deaths, and six (4.4%) had neurological deaths. In some patients, the cause of death involved more than one organ system. The definitive cause of death was not specified in 29 (21.2%) patients. Eighteen patients required repeat CABG surgery, with half of these patients re-operated within the same admission for CABG surgery (early redo). Among the patients that demised, only 2.2 and 1.5% were early and late redo cases. Valve replacement and CABG surgery were performed in the same surgical sitting in 85 (7.0%) patients. In these patients, the mortality rate was 27.0%.

**Fig. 3:**
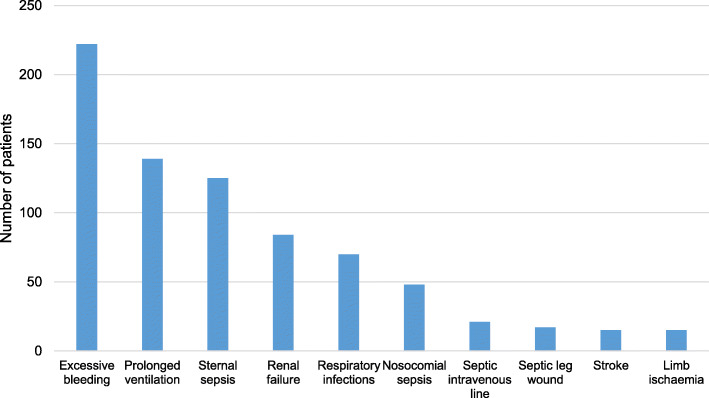
Perioperative complications in patients that underwent coronary artery bypass graft surgery

Perioperative bleeding requiring a blood transfusion or relook surgery was reported in 222 (18.2%), of which 109 went back to the operating theatre for haemostasis. However, some of these patients had repeated relooks. Mechanical ventilation exceeding 48 h was documented in 139 (11.4%) patients, and renal failure was identified as a complication of CABG in 84 (6.9%) patients. The remaining perioperative complications in patients that underwent CABG are summarised in Fig. 3. Relook operations were performed in 256 patients, with some patients sent back to the operating theatre more than once, resulting in a cumulative total of 496 relooks between year 2000 and 2017.

In the entire cohort, 176 (14.4%) patients had sepsis. The median body mass index in patients with and without sepsis was 27.8 (IQR: 24.9–32.4) vs. 27.5 (24.2–30.7) kg/m^2^, *p* = 0.1260. Among the patients with sepsis, 44.9% were smokers and 7.4% had peripheral vascular disease. The most frequent sepsis site was the midline sternotomy incision, reported in 125 (10.3%) patients. Deep sternal wounds were reported in 16 (1.3%) and superficial sternal wounds in 36 (2.9%). The sternal wounds were not characterised in the rest of the patients. Patients with sternal wound infections had a prolonged hospital stay of 23.5 (IQR: 19–37) vs. 13 (IQR: 11–17) days, *p* = 0.000, a higher median EuroSCORE II of 2.5 (IQR: 1.1–4.5) vs. 1.6 (0.9–3.3), *p* = 0.001 and a longer duration of cardiopulmonary bypass time of 144 (IQR: 118–182) vs. 138 (IQR: 114–166) minutes, *p* = 0.014.

**Fig. 4:**
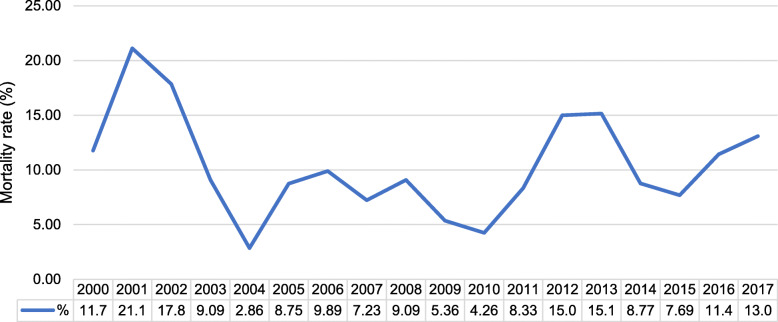
Mortality rate trends after coronary artery bypass graft surgeries between January 2000 and December 2017

Over the 17 years, the all-cause mortality rate ranged between 2.9 and 21.1%, with the highest mortality rate observed in the year 2001 (Fig. 4). When evaluating predictors of in-hospital all-cause mortality, univariable logistic regression analysis showed a linear relationship between mortality and the following variables: advanced age, diabetes mellitus, dyslipidaemia, smoking, New York Heart Association (NYHA) functional class II-IV, a poor left ventricular ejection fraction (LVEF), pre and post intra-aortic balloon pump insertion, a prolonged cardiopulmonary bypass (CPB) time and a high EuroSCORE II. However, a high EuroSCORE II and a prolonged CPB time were the only independent predictors of all-cause mortality in the multivariable logistic regression model (Table 2).

**Table 2:** Univariable and multivariable logistic regression analysis for predictors of in-hospital mortality

	Linear regression			Multivariable regression		
	OR	*p-*value	CI	OR	*p-*value	CI
Age	1.04	0.000	1.02–1.06			
Diabetes	1.43	0.054	0.99–2.06			
Dyslipidaemia	0.43	0.000	0.30–0.62			
Smoking	0.55	0.001	0.38–0.80			
NYHA class II	2.32	0.000	1.46–3.68			
NYHA class III	3.00	0.000	1.69–5.30			
NYHA class IV	12.3	0.000	5.30–28.40			
IABP (preop)	2.80	0.000	1.95–4.01			
IABP (postop)	3.95	0.000	2.12–7.35	1.42	0.002	0.52–2.31
LVEF	0.95	0.000	0.94–0.96			
CPB time (min)	1.01	0.000	1.01–1.02	0.01	0.000	0.00–0.02
EuroSCORE II	1.21	0.001	1.16–1.27	0.15	0.000	0.09–0.22

*CPB* Cardiopulmonary bypass; *CI* Confidence interval; *IABP* Intra-aortic balloon pump; *EuroSCORE* European System for Cardiac Operative Risk Evaluation; *LVEF* Left ventricular ejection fraction; *NYHA* New York Heart Association; *OR* Odds ratio

## Discussion

The risk of mortality in patients subjected to CABG surgery depends on the patients’ age, comorbidities, physiological functional reserve, degree of left ventricular dysfunction, the surgeons’ experience, and the hospital procedure volume [8–10]. The operative all-cause mortality rate in our hospital was 11.2%. Our mortality rate is significantly higher compared to that reported from other developing and developed countries. Data obtained from 1,145,285 patients referred for CABG in the United States of America (USA), from 1989 to 2004, showed a decline in mortality rate from 5.5 to 3.06%, irrespective of the presence of comorbidities [11]. A review of 17,335 CABG surgeries performed in Spain between 2013 and 2015 demonstrated a crude mortality rate of 5% [12]. Similarly, Swart et al. analysed outcomes post CABG in a South African cardio-thoracic private practice and reported a mortality rate of 3% [13].

Numerous clinical factors may have contributed to a higher mortality rate in our hospital. The patients in our cohort were older than those demonstrated in other studies, with a mean age of 60 years, while the mean age in the group of patients who demised was 63 years. Also, there may be a modest relationship between the surgical experience and the mortality rate. This hypothesis is argued by Peterson et al., who found that the surgeons’ experience was a predictor of mortality, independent of hospital volume, and the highest mortality rates were observed when patients were treated at low-volume hospitals by low-experience surgical teams, defined as performing 10 to 85 CABG surgeries per annum [14, 15]. In our hospital, the number of CABG surgeries was generally under 100 procedures per annum, except for the year 2017, where 107 CABG surgeries were performed. It is worth noting that although fewer CABG surgeries were performed in our hospital, most of our cardiothoracic surgeons are reasonably experienced as they also perform CABG surgeries in private hospitals.

Common postoperative complications associated with CABG reported in the literature are death, myocardial infarction, cardiac arrhythmia, stroke, wound infection, renal dysfunction, and bleeding requiring transfusion or repeat relook surgery [16]. In our study, 11.4% of patients required ventilation for more than 48 h, and the stroke rate was 1.2%. A similar rate of complications was reported in a study involving 36,588 patients after isolated CABG surgery, where 9% of patients required prolonged ventilation (> 24 h), and 1.2% experienced a stroke [17].

In our study, 1.3% of patients had deep sternal wound infections. This is a relatively high rate when compared to other studies. In larger cohorts, only 0.1% of patients had deep sternal wound infections [17]. Most data suggest that sepsis development following CABG portends a higher risk of mortality and a longer hospital stay [18–20]. In our cohort, patients with sternal wound infections were hospitalised for approximately 3 weeks.

In contrast, those without sternal wound infections had a median duration of hospitalisation of approximately 2 weeks. Variables associated with an increased risk of developing sepsis after CABG are a raised body mass index (BMI), poor preoperative glycaemic control, smoking, peripheral vascular disease, prolonged duration of cardiac bypass, and repeat or relook surgery [20]. In our study, the CPB time differed significantly between survivors and non-survivors (137 vs. 162 min) and independently predicted mortality (*p* = 0.000). Santos et al. studied 1628 patients post CABG surgery and found a median CPB time of 94 min. Similar to our findings, a longer CPB time predicted mortality [21].

Risk prediction models have been developed as tools for patient risk stratification. Widely-used risk models in patients planned for cardiac surgery include the Society of Thoracic Surgeons (STS) and the EuroSCORE II [22–24]. Although not externally validated in our population, the STS score and the EuroSCORE II are utilised in our hospital for risk assessment before CABG. The EuroSCORE II, revised in 2011, uses cardiac-specific and procedure-based variables to predict the patient’s risk of mortality following cardiac surgery. Studies reporting actual and predicted mortality risk categorize the EuroSCORE II into low (score between 0.17 and 0.80), intermediate (score between 0.81 and 2.02), high (score between 2.03 and 4.11), and very high (scores between 4.14 and 47.60) categories [25]. In this study, the EuroSCORE II independently predicted mortality and was significantly higher in the group of patients that demised.

### Limitations

Our study had several limitations. The study’s retrospective nature limited our access to pertinent clinical data, as some records from the earlier years were incomplete. Only complications that occurred during the same admission as the CABG surgery were reviewed, and complications requiring readmission at a later stage were not analysed. Another limitation was a paucity of clinical information in the records of patients transferred from other hospitals within the referral network. In these instances, the clinical data was gathered from the referral letters and angiography reports.

We noted a variation in the mortality rate during the study period but could not account for this variation due to the study’s retrospective nature. Furthermore, we identified a high rate of sternal wound infections but could not describe the organisms causing the infections in all patients with sepsis, nor define the treatment plan utilised. Despite these limitations, this retrospective review of perioperative complications in patients subjected to CABG surgery in a state-sector, tertiary academic hospital, adds more insights into locally relevant predictors of mortality and reports on the crude mortality rate over 17 years.

## Conclusions

In our study, the perioperative mortality rate was 11.2%, with most deaths attributed to cardiac complications. Excessive bleeding was the most frequent perioperative complication, reported in 18.2% of patients. Our mortality rate is significantly higher than the mortality rates reported in other developed and developing countries. To better understand the factors driving this high mortality rate, a prospective outcomes registry has been initiated and will inform on the contemporary mortality rate.

## Data Availability

The dataset used and analysed during the current study are available from the corresponding author on reasonable request.

## Abbreviations

CABG: Coronary artery bypass graft
CAD: Coronary artery disease
CMJAH: Charlotte Maxeke Johannesburg academic hospital
COPD: Chronic obstructive pulmonary disease
CPB: Cardiopulmonary bypass
eGFR: Estimated glomerular filtration rate
EuroSCORE: European system for cardiac operative risk evaluation
ICU: Intensive care unit
LVEF: Left ventricular ejection fraction
NYHA: New York Heart Association
PVD: Peripheral vascular disease
STS: Society of thoracic surgeons
USA: United States of America
